# sFlt-1/PlGF Ratio in the Prediction of Preeclampsia in Pregnant Women With Diabetic Kidney Disease

**DOI:** 10.1155/jdr/3987453

**Published:** 2025-06-06

**Authors:** Jakub Kornacki, Ewa Wender-Ozegowska, Daniel Boroń, Urszula Mantaj, Przemysław Wirstlein, Paweł Gutaj

**Affiliations:** Department of Reproduction, Poznan University of Medical Sciences, Poznan, Poland

**Keywords:** angiogenic imbalance, diabetes, diabetic kidney disease, placenta, preeclampsia, proteinuria

## Abstract

**Objectives:** The objective of the study is to evaluate the potential role of the sFlt-1/PlGF ratio in predicting preeclampsia (PE) in pregnant women with diabetic kidney disease (DKD) during the second and third trimesters of pregnancy.

**Study Design:** This study included 102 patients with DKD. All participants had a history of pregestational diabetes of at least 20 years' duration and/or onset before the age of 10 or pregestational diabetes complicated by hypertension or diabetic retinopathy (classified as D, R, or F according to White's classification). All patients exhibited elevated urinary protein loss (30–299 mg/24 h) or overt proteinuria (≥ 300 mg/24 h) during the first trimester of pregnancy. All participants were treated with intensive insulin therapy, either via multiple daily insulin injections (MDIs) or continuous subcutaneous insulin infusion (CSII). Serum levels of sFlt-1, PlGF, and the sFlt-1/PlGF ratio were assessed at both the 20th and 30th weeks of gestation.

**Main Outcome Measures:** The serum levels of sFlt-1, PlGF, and the sFlt-1/PlGF ratio during the second and third trimesters were compared between women who developed PE and a control group without PE.

**Results:** In DKD patients who developed PE, serum sFlt-1 levels were significantly higher, while PlGF levels were significantly lower, compared to those who did not develop PE. The sFlt-1/PlGF ratio was also markedly elevated in the PE group compared to controls during both the second and third trimesters. Increased sFlt-1 levels, decreased PlGF levels, and an elevated sFlt-1/PlGF ratio were significant predictors of PE development at both 20 and 30 weeks of gestation. The sFlt-1/PlGF ratio demonstrated a sensitivity of 72.7% and specificity of 75.7% at 20 weeks (sFlt-1/PlGF cut-off > 10.1) and a sensitivity of 84.6% and specificity of 82.0% at 30 weeks for predicting PE (sFlt-1/PlGF cut-off > 37). The positive predictive values were 47.1% and 50.0%, while the negative predictive values were 90.3% and 96.0% at 20 and 30 weeks, respectively.

**Conclusions:** The assessment of antiangiogenic and proangiogenic markers, particularly the sFlt-1/PlGF ratio, appears to be a valuable tool for predicting PE in patients with long-lasting diabetes complicated by DKD.

## 1. Introduction

Among different risk factors of preeclampsia (PE), pregestational diabetes mellitus (DM), especially long-term DM, is the most important [1, 2]. The risk of PE is exceptionally high in women with pregestational DM complicated by angiopathy, which encompasses hypertension, retinopathy, and nephropathy [3, 4]. Historically, the incidence of PE in women with diabetic kidney disease (DKD) was as high as 40%–60% [5, 6]. However, with advancements in insulin pump therapies, modern glucose monitoring techniques, and more effective preventative measures, the incidence of PE in this group has significantly declined [4, 7].

Managing DKD patients during pregnancy, particularly in the context of PE development, remains highly challenging. A primary obstacle is the overlap of clinical symptoms between PE and DKD, including hypertension and proteinuria. These markers of kidney dysfunction, often present as early as the first trimester in DKD patients, complicate the subsequent diagnosis of PE [8]. A good, practical approach to the diagnosis of PE in women with DKD was proposed by Wiles et al. [9]. However, it may still be difficult to differentiate PE from the signs of exacerbation of DKD.

Various markers and factors have historically been identified as determinants of PE in women with pregestational DM and DKD. These include hypertension, the degree of proteinuria—particularly in the first trimester—serum creatinine levels, creatinine clearance (CC) during the third trimester, and triglyceride (TG) levels [3, 4, 7].

Currently, angiogenic factors, particularly soluble fms-like tyrosine kinase 1 (sFlt-1) and placental growth factor (PlGF), as well as the sFlt-1/PlGF ratio, are regarded as the most valuable markers for PE [1, 10]. These markers are increasingly utilized in clinical practice, not only for predicting PE but also for ruling in or ruling out the disease [10]. Their utility appears especially significant in patients with DKD, where diagnosing PE is often complex and challenging.

Another intriguing aspect of PE in women with long-standing pregestational DM and DKD is the underlying pathophysiology. Given the heterogeneity of PE's pathogenesis, it is important to explore whether the two-stage model of the disease—characterized by impaired placental perfusion—also contributes to the development of PE in patients with DKD.

The primary aim of this study was to assess the role of placental dysfunction, including impaired placental perfusion, in the pathophysiology of PE in women with DKD. The secondary aim was to evaluate the potential role of the sFlt-1/PlGF ratio in predicting PE in this high-risk population.

## 2. Material and Methods

### 2.1. Subjects

This prospective study included 102 patients with DKD who were treated between 2016 and 2023 at the Department of Reproduction, Poznan University of Medical Sciences, Poland, a tertiary care center for diabetes in pregnancy. Participants had pregestational diabetes lasting at least 20 years and/or with an onset before the age of 10 or diabetes complicated by hypertension or diabetic retinopathy (classified as D, R, or F according to White's classification). All patients exhibited elevated urinary protein loss (30–299 mg/24 h) or overt proteinuria (≥ 300 mg/24 h) during the first trimester of pregnancy.

Patients with Type 1 diabetes were referred to the department upon confirmation of pregnancy. Optimal metabolic control, defined as glycosylated hemoglobin (HbA1c) < 6.5% in the first trimester and < 6.0% in the second and third trimesters, was targeted in accordance with Polish recommendations [11].

All participants underwent at least three planned, short-stay hospital admissions: during the first trimester, at midpregnancy (20–22 weeks), and at 30–32 weeks of gestation. Patients requiring more intensive monitoring were admitted more frequently, with the last hospitalization occurring no later than 34 weeks. Between hospital admissions, patients attended weekly or biweekly outpatient visits for regular check-ups.

### 2.2. PE Diagnosis

PE was diagnosed in patients without preexisting chronic hypertension using the following criteria [1]:

• Blood pressure: systolic ≥ 140 mmHg or diastolic ≥ 90 mmHg on two occasions after 20 weeks of gestation, along with at least one of the following:
  o. Proteinuria (≥ 300 mg/24 h or > 100% increase in proteinuria in proteinuric patients)
  o. Serum creatinine > 1 mg/dL (> 90 *μ*mol/L) or a > 50% increase within 7 days
  o. Elevated transaminase levels (> 40 IU/L)
  o. Neurological complications (e.g., eclampsia, altered mental status, stroke, clonus, severe headache, or visual scotomata)
  o. Hematological complications (e.g., thrombocytopenia < 150 G/L, disseminated intravascular coagulation, or hemolysis)
  o. Uteroplacental dysfunction (e.g., fetal growth restriction (FGR) or abnormal Doppler indices) [12]

In patients with chronic hypertension, PE was diagnosed following the onset of severe hypertension (systolic BP > 160 mmHg or diastolic BP > 110 mmHg) after 20 weeks of gestation or the need to escalate antihypertensive therapy, accompanied by at least one additional criterion as outlined above, excluding uteroplacental dysfunction according to ISSHP (International Society for the Study of Hypertension in Pregnancy) [1].

FGR was diagnosed based on Delphi consensus criteria [12]. Fetal Doppler assessments were conducted for estimated fetal weight (EFW) or abdominal circumference (AC) below the 10th percentile after 20 weeks. Pulsatility index (PI) was measured in the umbilical artery, middle cerebral artery (MCA), and uterine arteries. The cerebroplacental ratio (CPR) was calculated from 32 weeks onward. Sonographic and Doppler evaluations were performed using the S10 Expert ultrasound system (GE Healthcare).

Large for gestational age (LGA) newborns were diagnosed when the birth weight was above the 90th percentile by using age- and sex-specific regional growth charts.

Fifty-two patients (51%) received 150 mg of aspirin daily from 12 to 16 weeks of gestation until 36 weeks or the onset of PE, following recommendations from the Polish Society of Hypertension, Polish Cardiac Society, and Polish Society of Gynecologists and Obstetricians [13]. The remaining 50 patients (49%) did not receive aspirin during pregnancy.

### 2.3. Monitoring of Laboratory and Clinical Measurements

All participants underwent intensive insulin therapy using either multiple daily injections (MDIs) or continuous subcutaneous insulin infusion (CSII). Daily glucose monitoring included fasting, pre- and postprandial values twice weekly, with 5–6 measurements on other days.

Laboratory tests conducted each trimester included the following:

1. HbA1c levels
2. Serum TG
3. 24-h urinary protein loss
4. CC
5. Serum creatinine levels
6. Transaminase levels
7. Platelet counts

At 20 and 30 weeks of gestation, serum levels of sFlt-1 and PlGF were assessed, and the sFlt-1/PlGF ratio was calculated.

Critical clinical findings monitored in the studied group included fetal biometry, a daily dose of insulin, methods of insulin delivery (insulin pens or insulin pump), and changes in patients' body weight.

### 2.4. Sample Collection and Analysis

Blood samples for all routine analyses were collected after overnight fasting and immediately transported to the accredited laboratory of the university hospital, which has received ISO 9000 quality management certification. HbA1c in whole blood was determined using the turbidimetric inhibition immunoassay (TINIA), Tina-quant Hemoglobin A_1c_ II test in a Cobas c311 analyzer (Roche Diagnostics). The normal range is 29–42 mmol/mol (4.8%–6.0%) for a nonpregnant population. We used three HbA1c values: the first was measured in the first trimester or at first admission, the second was measured in the second trimester between 18 and 22 gestational weeks, and the third was measured before delivery. For calculations, we used the average value if two HbA1c values were measured in the analyzed period of pregnancy.

To determine sFlt-1 and PlGF concentration, 7.5 mL of venous blood was collected from all the studied patients. After centrifugation (2000×*g*), the obtained serum was frozen at −20°C for further assessment. sFlt-1 and PlGF levels were determined by enzyme-linked immunosorbent assay (ELISA) kits (DRG Instruments GmbH, Germany). The assays were performed according to the manufacturer's instructions. Plate reading was conducted using an MRX reader (Dynex Technology, Chantilly, Virginia, United States) at *λ* = 450 nm with corrections at 570 nm.

### 2.5. Ethics

Written informed consent was obtained from all participants. The study protocol was reviewed and approved by the Poznan University of Medical Sciences Bioethics Committee (No. 291/21).

### 2.6. Statistical Analysis

SigmaStat Version 3.5 software (Systat Software Inc., Point Richmond, California, United States) was used for statistical analysis. The Kolmogorov–Smirnov test was used to verify normality. Group comparisons were analyzed using Student's *t*-test for variables with a normal distribution and the Mann–Whitney rank sum test for variables with a nonnormal distribution. For nonquantitative characteristics, groups were compared using the chi-square test. Logistic regression and receiver operating characteristic (ROC) curve analyses were performed using MedCalc for Windows, Version 23.0.5 (MedCalc Software, Mariakerke, Belgium). Simple logistic regression was used to search for factors associated with PE. For multiple variable regression analysis, we used a logistic regression model with a stepwise forward approach. Variables were entered into the model based on their univariate significance (*p* < 0.05), and adjustments were made for chronic hypertension and baseline proteinuria, as these were identified as clinically relevant confounders. The inclusion of adjustment variables was evaluated using likelihood ratio tests. To control for the multiplicity of testing, we applied the Benjamini–Hochberg false discovery rate (FDR) correction. Statistical significance was defined as *p* < 0.05 (two-sided).

## 3. Results

Among the 102 studied patients, at the time of inclusion into the study, proliferative retinopathy was diagnosed in 26 women (26.5%), proteinuria in 17 (16.7%) patients, and chronic hypertension in 22 (21.6%) patients.

PE was diagnosed in 22 patients (27.5%) with DKD. The mean gestational age at diagnosis of PE was 33 + 0 weeks. Two patients who developed PE delivered before 30 weeks of gestation, at 28 and 29 weeks, so they did not have the second assessment of sFlt-1 and PlGF levels at 30 weeks. Clinical characteristics of the patients who developed PE and the women in whom PE was not found are presented in Table 1.

Table 2 presents the pregnancy outcome in both groups of patients.

Patients who developed PE exhibited significantly higher HbA1c levels during the second trimester, as well as elevated creatinine concentrations in both the first and second trimesters, compared to those in the control group. Furthermore, the third-trimester CC values were significantly lower in the PE group. Proteinuria levels were consistently and significantly higher across all trimesters in patients who developed PE compared to those who did not.

The selected laboratory parameters for both groups are summarized in Table 3.

At both 20 and 30 weeks of gestation, the level of sFlt-1 was significantly higher, while the level of PlGF was significantly lower in patients who developed PE compared to the control group. The sFlt-1/PlGF ratio was markedly elevated in the study group compared to women who did not develop PE at both gestational time points.

The levels of sFlt-1, PlGF, and the sFlt-1/PlGF ratios in both groups during the second and third trimesters are detailed in Table 4.

The sFlt-1/PlGF ratio demonstrated a sensitivity of 72.7% and specificity of 75.7% at 20 weeks (sFlt-1/PlGF cut-off > 10.1) and a sensitivity of 84.6% and specificity of 82.0% at 30 weeks for predicting PE (sFlt-1/PlGF cut-off > 37). The positive predictive values were 47.1% and 50.0%, while the negative predictive values were 90.3% and 96.0% at 20 and 30 weeks, respectively (Table 5).

Chronic hypertension and baseline proteinuria were selected as adjustment variables based on their clinical relevance and prior evidence suggesting their strong association with PE in women with DKD [4, 7].

Multiple variable regression analysis focusing on the sFlt-1/PlGF ratio as a predictor of PE, adjusted for chronic hypertension and daily urinary protein excretion, is presented in Table 6. Neither chronic hypertension nor baseline proteinuria significantly altered the association between the sFlt-1/PlGF ratio and the development of PE.

**Figure 1:**
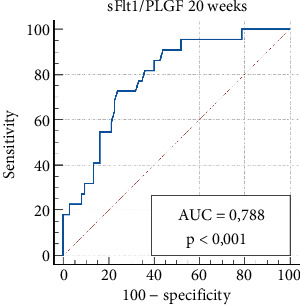
Receiver operating characteristic (ROC) analysis of the sFlt1/PlGF ratio at 20 weeks of gestation for predicting the development of preeclampsia. The criterion value, with a sensitivity of 72.7% and specificity of 76.0%, was greater than 10.1.

**Figure 2:**
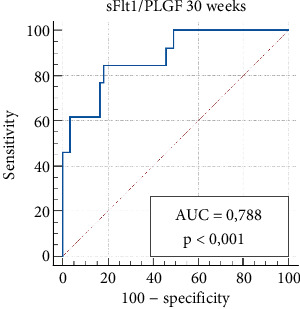
Receiver operating characteristic (ROC) analysis of the sFlt1/PlGF ratio at 30 weeks of gestation for predicting the development of preeclampsia. The criterion value, with a sensitivity of 84.6% and a specificity of 82.0%, was greater than 37.

Figures 1 and 2 illustrate ROC curves for the sensitivity and specificity of the sFlt-1/PlGF ratio in predicting PE at 20–30 weeks of gestation, respectively.

The proportion of patients taking aspirin was not statistically different between the groups with and without PE (56.5% vs. 63.6%, *p* = 0.3924).

## 4. Discussion

The frequency of PE in our cohort of patients with long-standing pregestational diabetes complicated by chronic kidney disease (CKD), with or without other angiopathic complications, was 27.5%. This is notably lower than the rates reported in older studies, such as those by Ekbom et al. [14], Young et al. [6], and Klemetti et al. [5]. For example, Ekbom et al. [14] documented PE in 42% and 64% of patients with Type 1 diabetes and microalbuminuria or nephropathy, respectively. This reduction likely reflects advancements in the management of diabetes, including the widespread use of insulin pumps and improved prophylaxis and diagnostic strategies for PE.

Our findings confirm prior observations that the risk of PE in women with DKD is significantly associated with the degree of proteinuria across all trimesters, chronic hypertension, and proliferative retinopathy. These findings align with those of Vestgaard et al. [7] and Nielsen et al. [15], the latter of whom identified the urine albumin-to-creatinine ratio as a key predictor of PE in this population.

The other possible predictors of PE development in women with DKD found in our study were (1) serum creatinine level in the first and third trimester of pregnancy, (2) CC in the third trimester, and (3) HbA1c in the second trimester.

It confirms the results of previous studies, mainly on HbA1c [3, 5, 15–17]. Characteristically, in the survey by Gutaj et al. [3] and Cavero–Redondo et al. [16], a stronger association of PE risk with second-trimester HbA1c levels compared to first-trimester values was found.

While numerous biomarkers have been investigated for their predictive value in PE, such as TGs, leptin, adiponectin, and urinary neutrophil gelatinase-associated lipocalin (uNGALcc) [3, 18, 19], angiogenic and antiangiogenic factors, including the sFlt-1/PlGF ratio, have emerged as critical tools.

There are only a few studies on the prediction of PE in women with pregestational diabetes with the use of angiogenic and antiangiogenic factors, including sFlt-1/PlGF ratio [20–24]. However, none of these studies included patients with pregestational diabetes complicated by microalbuminuria or proteinuria as in our study. Moreover, in the study by Kelly et al. [20], microalbuminuria and proteinuria were among the exclusion criteria.

In one of the studies mentioned above [20] sFlt-1, PlGF, and sFlt-1/PlGF ratio were found to be predictive of PE only in the third trimester of pregnancy. The same conclusion was made by Provendier et al. [23], but they analyzed only the results of the ratio from 30 to 34 weeks. It is inconsistent with the results of the present study in which we found significant differences in all markers, including in the ratio, both at 20 and 30 weeks of gestation. It may be explained by the fact that even in the general population the decreased level of PLGF was found to be lower in the first trimester of pregnancy in women who developed PE [25]. At the same time, the increased sFlt-1/PlGF ratio usually is found already 5 weeks before the onset of PE [25]. Because we examined a particular group of patients with long-lasting diabetes complicated by different forms of angiopathy, we suspected the possible higher sensitivity of these markers in this group. It may be the consequence of a preexisting higher degree of microvascular pathology, including endothelial dysfunction and a higher degree of macroangiopathy. The latter may contribute to more disturbed placental perfusion, a primary pathophysiological phenomenon responsible for the increased production of sFlt-1.

Our results align closely with those of Holmes et al. [24]. The mentioned authors, in so far as the most extensive survey on that topic, showed increased values of sFlt-1 and sFlt-1/PlGF ratio and decreased concentration of PlGF at 26 weeks of gestation in patients with pregestational Type 1 diabetes who developed PE compared to those in whom PE was not diagnosed. However, this study group consisted mainly of patients with normoalbuminuria, whereas the material of our study included women with long-lasting DM complicated by different degrees of kidney disease.

Despite this difference, both studies underscore the clinical utility of sFlt-1/PlGF measurements in predicting PE during the second and third trimesters in women with Type 1 diabetes.

Of particular importance, we identified the sFlt-1/PlGF ratio at both 20 and 30 weeks and the PlGF level at 30 weeks as independent significant risk factors for PE in women with DKD. These findings emphasize the role of placental pathology, including abnormal development and impaired perfusion, as a key contributor to PE in this high-risk population, especially in cases of preexisting proteinuria and hypertension.

## 5. Conclusions

Besides showing the usefulness of anti- and angiogenic markers in women with pregestational diabetes and DKD in predicting PE, our results indicate the similar pathological pathway of the development of PE in these groups of patients as in the general population. In both cases, the two-stage model of the disease, which includes impaired placental perfusion and later maternal endothelial dysfunction, seems to be the main pathological phenomena in PE. However, our study indicates that women with pregestational diabetes and DKD may be particularly susceptible to both placental perfusion abnormalities and endothelial injury, potentially making them more prone to the development of PE.

This observation raises an interesting question: could these inherent vascular vulnerabilities explain why aspirin, a well-established prophylactic treatment for PE, is less effective in this high-risk group?

In our opinion, the use of the markers studied, including the sFlt-1/PlGF ratio, could be particularly valuable for monitoring patients with pregestational, complicated diabetes. Compared to some other novel predictors, these markers are widely available and commonly used in clinical practice, making them familiar to clinicians and easier to incorporate into routine care. Moreover, their levels can be easily monitored, providing practical utility for both diagnosis and management. The changes in these markers may also more accurately reflect the underlying pathophysiological mechanisms responsible for PE development in women with pregestational diabetes, suggesting that the process does not differ significantly from that in the general population.

## Figures and Tables

**Table 1:** Clinical characteristics of patients with DKD who developed PE and patients with DKD who were not diagnosed with PE.

**Variables**	**Preeclampsia (** **n** = 22**)**	**No preeclampsia (** **n** = 80**)**	**p** ** value**
Age at conception (years)	32 ± 6	29 ± 5	0.1249
Primiparity	15 [70]	66 [83]	0.4380
Age at diabetes diagnosis (years)	11 [4–18]	9 [2–32]	0.2400
Duration of diabetes (years)	20 ± 6	17 ± 57	0.0670
Gestational week at 1st admission (weeks)	9 [5–24]	9 [5–24]	0.5009
Chronic hypertension	**10 [45]**	**10 [12]**	**0.0037**
Overt proteinuria at the onset of the study	**10 [45]**	**11 [14]**	**0.0024**
Proliferative retinopathy at the onset of the study	**15 [70]**	**20 [25]**	**0.0001**
Pregestational BMI (kg/m^2^)	24.0 ± 3.7	25.0 ± 4.7	0.3675
Weight gain during pregnancy (kg)	10.1 ± 5.9	10.7 ± 6.3	0.6892
BMI prior to delivery (kg/m^2^)	27.7 ± 3.9	28.6 ± 4.5	0.2311
Insulin taken before pregnancy (IU/day)	37.7 ± 13.0	34.6 ± 12.8	0.2966
Insulin taken in the 3rd trimester of pregnancy (IU/day)	52.5 ± 20.0	55.2 ± 19.6	0.5646

*Note:* Data presented as *n* [%], mean ± SD, or median (range). Bold signifies significant differences.

Abbreviations: BMI, body mass index; IU, insulin unit.

**Table 2:** Pregnancy outcome in patients with DKD who developed PE and patients with DKD who were not diagnosed with PE.

**Variables**	**Preeclampsia (** **n** = 22**)**	**No preeclampsia (** **n** = 80**)**	**p** ** value**
Neonatal birth weight (g)	2597 ± 888	3449 ± 583	**< 0.0001**
Gestational age at delivery (week)	**35 [28**–**38]**	**38 [30**–**39]**	**< 0.0001**
Cesarean section	22 (100)	66 (82.5)	0.2309
Emergency indications to caesarean section	7 (30)	15 (22)	0.6216
Fetal growth restriction (FGR)	**5 (22.7)**	**1 (1.2)**	**0.0001**
Large for gestational age (LGA)	2 (9.1)	20 (25)	0.1865

*Note:* Data presented as *n* [%], mean ± SD, or median (range). Bold signifies significant differences.

**Table 3:** Results of laboratory findings in patients with DKD who developed PE and in patients with DKD who were not diagnosed with PE.

**Variables**	**Preeclampsia (** **n** = 22**)**	**No preeclampsia (** **n** = 80**)**	**p** ** value**
HbA1c in the 1st trimester (%)	7.23 ± 1.45	6.84 ± 1.02	0.0817
HbA1c in the 2nd trimester (%)	6.10 ± 0.95	5.69 ± 0.64	**0.0143**
HbA1c 3rd trimester (%)	6.12 ± 0.71	5.94 ± 0.65	0.2672
Triglycerides in the 1st trimester (mg/dL)	66.6 [31.8–236.4]	68.05 [38.1–286.1]	0.5003
Triglycerides in the 2nd trimester (mg/dL)	117.2 [83.8–295.7]	132.6 [65.8–502.7]	0.4942
Triglycerides in the 3rd trimester (mg/dL)	245.84 ± 84.15	271.48 ± 90.56	0.2215
Proteinuria in the 1st trimester (g/day)	**0.28 [0.11**–**5.73]**	**0.19 [0**–**2.13]**	**0.0009**
Proteinuria in the 2nd trimester (g/day)	**0.35 [0.12**–**4.9]**	**0.18 [0.08**–**5.45]**	**0.0043**
Proteinuria in the 3rd trimester (g/day)	**1.63 [0.26**–**7.87]**	**0.22 [0.08**–**6.24]**	**< 0.0001**
Creatinine clearance in the 1st trimester (mL/min)	127.91 ± 50.55	134.28 ± 42.42	0.5560
Creatinine clearance in the 2nd trimester (mL/min)	116.73 ± 50.47	137.42 ± 40.54	0.0516
Creatinine clearance in the 3rd trimester (mL/min)	97.85 ± 39.89	129.8 ± 42.12	**0.0040**
Serum creatinine in the 1st trimester (mg/dL)	0.70 ± 0.29	0.58 ± 0.14	**0.0094**
Serum creatinine in the 2nd trimester (mg/dL)	0.60 [0.42–1.64]	0.57 [0.37–1.32]	0.2604
Serum creatinine in the 3rd trimester (mg/dL)	**0.7 [0.5**–**1.81]**	**0.62 [0.28**–**2.72]**	**0.0224**
Alanine transaminase in the 3rd trimester (IU/L)	13.9 (6.3–108.9]	11.6 [5.3–32.9]	0.1740
Aspartate transaminase (IU/L) in the 3rd trimester	28.3 ± 22.3	10.7 ± 6.3	0.0630
Platelet count (×10^9^/L) in the 3rd trimester	212 ± 59	223 ± 58	0.4232

*Note:* Data presented as *n* [%], mean ± SD, or median (range). HBA1c, glycated hemoglobin. Bold signifies significant differences.

**Table 4:** The levels of sFlt-1 and PlGF and the values of the sFlt-1/PlGF ratio in both groups of patients at 20 and 30 weeks of gestation.

	**Preeclampsia (** **n** = 22**)**	**No preeclampsia (** **n** = 80**)**	**p** ** value**
sFlt-1 (ng/mL) at 20 weeks	2.37 [0.37–19.88]	0.87 [0.07–36.75]	**p** < 0.0001
sFlt-1 (ng/mL) at 30 weeks	6.86 [1.96–12.96]	1.84 [0.16–34.10]	**p** = 0.0001
PlGF (ng/mL) at 20 weeks	0.11 [0–0.54]	0.23 [0.01–1.28]	**p** = 0.0070
PlGF (ng/mL) at 30 weeks	0.03 [0–0.53]	0.36 [0.03–1.74]	**p** = 0.0001
sFlt-1/PlGF at 20 weeks	20.3 [1.8–823.2]	4.3 [0.4–121.4]	**p** < 0.0001
sFlt-1/PlGF at 30 weeks	178.2 [6.6–5183.2]	6.4 [0.4–198.9]	**p** < 0.0001

*Note:* Data presented as median (range). Bold signifies significant differences.

**Table 5:** Sensitivity, specificity, PPV, and NPV for predicting PE at 20th and 30th weeks of gestation.

**Parameter**	**20th week**	**30th week**
Sensitivity (%)	72.7	84.6
Specificity (%)	75.7	82.0
Positive predictive value (%)	47.1	50.0
Negative predictive value (%)	90.3	96.0

**Table 6:** Multiple variable regression analysis of factors focusing on the sFlt-1/PlGF ratio as a predictor of PE, adjusted for chronic hypertension and daily urinary protein excretion predictive for PE in women with DKD.

**Determinants of PE**	**Adjusted OR**	**95% CI**	**p** ** value**
sFlt-1/PlGF at 20 weeks adjusted for the presence of chronic hypertension	1.0193	1.0034–1.0356	**0.0175**
sFlt-1/PlGF at 30 weeks adjusted for the presence of chronic hypertension	1.0173	1.0034–1.0314	**0.0148**
sFlt-1/PlGF at 20 weeks adjusted for 24-h urinary protein excretion in the first trimester	1.0159	1.0014–1.0307	**0.0320**
sFlt-1/PlGF at 30 weeks adjusted for 24-h urinary protein excretion in the first trimester	1.0211	1.0032–1.0392	**0.0206**
PlGF (ng/mL) at 20 weeks adjusted for the presence of chronic hypertension	0.0415	0.0012–1.4003	**0.0763**
PlGF (ng/mL) at 30 weeks adjusted for the presence of chronic hypertension	0.0001	0.0000–0.0960	**0.0091**
PlGF (ng/mL) at 20 weeks adjusted for 24-h urinary protein excretion in the first trimester	0.0605	0.0016–2.2638	0.1291
PlGF (ng/mL) at 30 weeks adjusted for 24-h urinary protein excretion in the first trimester	0.0000	7.0903E − 15 to 0.0403	**0.0169**

*Note:* Bold signifies significant differences.

## Data Availability

We inform that data supporting the findings of the study are available on request from the corresponding author. The data are not publicly available due to privacy or ethical restrictions.

## Supporting Information

Supporting Information Additional supporting information can be found online in the Supporting Information section. The results of single variable regression analysis identifying factors predictive of PE in women with diabetic kidney disease (DKD) are presented in Table S1. Maternal age, duration of diabetes, parity, insulin delivery method (pump vs. multiple daily injections, MDI), sFlt-1 levels at 30 weeks, and first-trimester HbA1c were not significantly associated with increased odds of PE (data not shown in Table S1).
