# Supplementary material for: sFlt-1/PlGF Ratio in the Prediction of Preeclampsia in Pregnant Women With Diabetic Kidney Disease
Source: J Diabetes Res. 2025 Jun 6;2025:3987453. doi: 10.1155/jdr/3987453 (PMC12165755; doi:10.1155/jdr/3987453)
Supplement: Supporting Information — Additional supporting information can be found online in the Supporting Information section. The results of single variable regression analysis identifying factors predictive of PE in women with diabetic kidney disease (DKD) are presented in Table S1. Maternal age, duration of diabetes, parity, insulin delivery method (pump vs. multiple daily injections, MDI), sFlt-1 levels at 30 weeks, and first-trimester HbA1c were not significantly associated with increased odds of PE (data not shown in Table S1). [file 3987453.f1.docx]

Table S1

Single variable analysis of factors predictive for PE in women with DKD.

| **Determinants of PE** | **Crude OR** | **95% CI** | **p-value** | **FDR-adjusted p-value** |
| --- | --- | --- | --- | --- |
| sFlt-1/PlGF at 20 weeks | 1.0160 | 1.0027–1.0295 | **0.0182** | **0.0364** |
| sFlt-1/PlGF at 30 weeks | 1.0153 | 1.0037–1.0271 | **0.0098** | **0.0392** |
| sFlt-1 at 20 weeks, ng/ml | 1.1293 | 1.0023–1.2723 | **0.0457** | **0.0457** |
| PlGF at 20 weeks, ng/ml | 0.0250 | 0.0008–0.8180 | **0.0382** | **0.0437** |
| PlGF at 30 weeks, ng/ml | 0.0001 | 0.0000–0.1142 | **0.0102** | **0.0272** |
| HbA1c at 20 weeks, % | 2.1636 | 1.1353–4.1235 | **0.0190** | **0.0304** |
| Chronic hypertension (1-yes, 0-no) | 5.1923 | 1.7704–15.2283 | **0.0027** | **0.0216** |
| 24h urinary protein excretion | 9.8375 | 1.2388–78.1183 | **0.0306** | **0.0408** |

FDR-adjusted p-values were calculated using the Benjamini-Hochberg procedure to control for multiple testing. All predictors remain statistically significant (p < 0.05) after FDR correction.
